# Prevalence, causes, and behavioral and emotional comorbidities of acute symptomatic seizures in Africa: A critical review

**DOI:** 10.1002/epi4.12035

**Published:** 2017-01-24

**Authors:** Symon M. Kariuki, Amina Abubakar, Alan Stein, Kevin Marsh, Charles R. J. C. Newton

**Affiliations:** ^1^ KEMRI‐Wellcome Trust Research Programme Kilifi Kenya; ^2^ Department of Public Health Pwani University Kilifi Kenya; ^3^ Department of Psychiatry University of Oxford Oxford United Kingdom; ^4^ Nuffield Department of Medicine University of Oxford Oxford United Kingdom; ^5^ Alliance for Accelerating Excellence in Science in Africa African Academy of Sciences Nairobi Kenya

**Keywords:** Acute symptomatic seizures, Febrile seizures, Infants and young children, Behavioral and emotional problems, Epidemiology

## Abstract

Seizures with fever includes both febrile seizures (due to nonneurological febrile infections) and acute symptomatic seizures (due to neurological febrile infections). The cumulative incidence (lifetime prevalence) of febrile seizures in children aged ≤6 years is 2–5% in American and European studies, but there are no community‐based data on acute symptomatic seizures in Africa. The incidence of acute symptomatic seizures in sub‐Saharan Africa is more than twice that in high‐income countries. However, most studies of acute symptomatic seizures from Africa are based on hospital samples or do not conduct surveys in demographic surveillance systems, which underestimates the burden. It is difficult to differentiate between febrile seizures and acute symptomatic seizures in Africa, especially in malaria‐endemic areas where malaria parasites can sequester in the brain microvasculature; but this challenge can be addressed by robust identification of underlying causes. The proportion of complex acute symptomatic seizures (i.e., seizures that are focal, repetitive, or prolonged) in Africa are twice that reported in other parts of the world (>60% vs. ∼30%), which is often attributed to falciparum malaria. These complex phenotypes of acute symptomatic seizures can be associated with behavioral and emotional problems in high‐income countries, and outcomes may be even worse in Africa. One Kenyan study reported behavioral and emotional problems in approximately 10% of children admitted with acute symptomatic seizures, but it is not clear whether the behavioral and emotional problems were due to the seizures, shared genetic susceptibility, etiology, or underlying neurological damage. The underlying neurological damage in acute symptomatic seizures can lead not only to behavioral and emotional problems but also to neurocognitive impairment and epilepsy. Electroencephalography may have a prognostic role in African children with acute symptomatic seizures. There are significant knowledge gaps regarding acute symptomatic seizures in Africa, which results in lack of reliable estimates for planning interventions. Future epidemiological studies of acute symptomatic seizures should be set up in Africa.

**Key Points**

- This review provides the epidemiology of acute symptomatic seizures in Africa, compared with elsewhere in the world, and identifies a lack of community‐based studies of acute symptomatic seizures in Africa
- Falciparum malaria is the most important cause of acute symptomatic seizures in Africa, and the proportion of complex acute symptomatic seizures (i.e., seizures that are focal, repetitive, or prolonged) in Africa are twice that reported in other parts of the world (>60% vs. ˜30%)
- In Africa, children hospitalized with acute symptomatic seizures are at elevated risk of behavioral or problems and cognitive impairments, but it is unclear whether the poor outcomes reported in Africa were due to the seizures, shared genetic susceptibility, etiology, or underlying neurological damage

Seizures occurring with fever or in a febrile illness are usually referred to as febrile seizures or acute symptomatic seizures, and these are the most common seizure disorders in young children.^1^ The lifetime prevalence of febrile seizures in children aged ≤6 years is 2–5% in American and European studies.^2^, ^3^ There are, however, few epidemiological studies of acute symptomatic seizures in sub‐Saharan Africa; one epidemiological study in Tanzania estimated prevalence of febrile seizures at 2.1%.^4^ The incidence of acute symptomatic seizures in young children admitted to hospitals in Africa is in the excess of 1,000 per 100,000/year,^5^, ^6^ which is likely to be grossly underestimated because only a proportion of children with seizures are treated in the hospital.^7^

Falciparum malaria is the most important cause of acute symptomatic seizures in malaria‐endemic countries.^8^ Most seizures in malaria occur when the child is afebrile,^9^ suggesting the underlying mechanisms may be different from that of febrile seizures in high‐income countries. Falciparum malaria can cause seizures because parasite‐infected erythrocytes sequester in the brain, causing diffuse brain damage that may manifest as acute symptomatic seizures.^10^

### Outline of the review

In this review, the terms “febrile seizures” and “acute symptomatic seizures” are used separately as appropriately defined below. The review outlines the prevalence of acute symptomatic seizures and febrile seizures reported in the literature, with an emphasis on Africa, where falciparum malaria is an important cause of acute symptomatic seizures. The paper further discusses the pathophysiology, phenotypes, and behavioral and emotional consequences of acute symptomatic seizures and febrile seizures, comparing the situation in Africa with other parts of the world. The underlying neurological damage that could explain the association between acute symptomatic seizures and behavioral and emotional problems may also be responsible for risk of epilepsy and neurocognitive impairments following these seizures, so these outcomes, too, are briefly covered in the review. The paper concludes by identifying the knowledge gaps of acute symptomatic seizures in Africa, particularly the striking lack of epidemiological studies, and provides the future directions of research of acute symptomatic seizures in Africa and other low‐income settings.

### Definitions of acute symptomatic seizures and febrile seizures

Both the National Institutes of Health (NIH)^11^, ^12^ and International League Against Epilepsy (ILAE)^13^ define febrile seizures as seizures associated with fever or a febrile illness, although they differ slightly on age limit and exclusion criteria (Table 1). However, this definition does not exclude febrile seizures in children with developmental delays unrelated to the febrile illness.^14^ The definition for febrile seizures excludes children with previous unprovoked seizures and neonatal seizures, and those with febrile infections that affect the central nervous system such as meningitis, encephalitis, and falciparum malaria. Acute symptomatic seizures are defined as seizures occurring in close temporal association with an acute central nervous system insult, which may be metabolic, toxic, structural, infectious, or related to inflammation.^2^

**Table 1:** Definition of febrile seizures according to International League Against Epilepsy (ILAE) and National Institutes of Health (NIH)

ILAE^13^	NIH^12^
A seizure occurring in childhood	Event in infancy or childhood
Occurs between 1 month and 5 years	Occurs between 3 months and 5 years
Associated with a febrile illness	Associated with fever
Excludes CNS infections, neonatal seizures, and acute symptomatic seizures	Excludes intracranial infections or seizures with defined causes

CNS, central nervous system.

### Challenges with definitions of acute symptomatic seizures in Africa

In sub‐Saharan Africa, falciparum malaria is the most common cause of seizures with fever,^5^, ^6^, ^8^ which should be referred to as acute symptomatic seizures because *Plasmodium falciparum*–infected erythrocytes sequester in the brain vessels. Falciparum malaria is the most important cause of acute symptomatic seizures in malaria‐endemic countries.^8^ Most seizures in malaria occur when the child is afebrile,^9^ suggesting the underlying mechanisms may be different from those of febrile seizures in high‐income countries. Falciparum malaria can cause acute symptomatic seizures because parasite‐infected erythrocytes sequester in the brain, causing diffuse brain dysfunction that may manifest as acute symptomatic seizures.^10^ Even the acute symptomatic seizures in otherwise nonneurological and uncomplicated malaria present with complex phenotypes (focal, prolonged, or repetitive) in 84% of children, with up to 54% occurring in nonfebrile temperatures,^9^ suggesting they are not febrile seizures per se but an extended spectrum of acute symptomatic seizures. Quick resolution of fever in those with acute symptomatic seizures (compared to those without seizures)^6^ suggests that seizures persisting or continuing after the cessation of fever are probably attributable to malarial disease process rather than the fever per se.^8^ For this reason, most acute symptomatic seizures in Africa are often misclassified as febrile seizures.

In a Benin study of hospitalized children, some of what was described as febrile seizures in children aged 5–60 months could have been acute symptomatic seizures because falciparum malaria accounted for 33% of all cases.^15^ There is a need to establish the criteria for defining acute symptomatic seizures and febrile seizures caused by falciparum malaria in endemic areas of sub‐Saharan Africa. Another study in Nigeria found intracranial infections such as malaria and meningitis in 38% of children admitted with seizures,^16^ although they inappropriately referred to these as febrile seizures. Three studies in Kenya used the term “acute symptomatic seizures,”^5^, ^6^, ^8^ as recommended by ILAE^2^ and found malaria as an important cause of seizures. One of these studies^8^ attempted to define febrile seizures by excluding those with parasitemia and meningitis.

## Addressing Challenges with Definitions of Acute Symptomatic Seizures and Febrile Seizures in Africa

Majority of seizures with fever occurring in malaria‐endemic areas of Africa should be suspected to be acute symptomatic seizures because perinatal complications and intracranial infections such as falciparum malaria, viral encephalitis, and bacterial meningitis are common in the community.^17^, ^18^ These causes can directly perturb the brain function and should be excluded from defining febrile seizures according to the ILAE guidelines.^2^ Separation of febrile seizures from acute symptomatic seizures in Africa should be informed by comprehensive investigations that rule out intracranial causes, as was attempted in a Kenyan hospital study,^8^ although this is logistically challenging to implement in community‐based studies. Most acute symptomatic seizures in the communities of Africa are not treated at the hospital,^7^ and so causes are never identified, making it difficult to distinguish febrile seizures from acute symptomatic seizures.

The following approaches can be utilized to improve definitions for seizures with fever occurring in communities in African countries and other low‐ and middle‐income countries (LMICs). Clinicians and researchers should follow ILAE guidelines for definition of acute symptomatic seizures and febrile seizures. Children with seizures with fever in the community can be tested with rapid diagnostic tests built from protein markers specific to falciparum malaria and invasive bacterial infections,^19^, ^20^ although these may be limited in excluding previous infections. Conducting community surveys of acute symptomatic seizures in seasons with low transmission of malaria and other invasive infections may improve identification of febrile seizures and acute symptomatic seizures. In poor regions with limited resources, the World Health Organization (WHO) clinical algorithms on integrated management of childhood infections may be modified to help identify underlying causes of acute symptomatic seizures not treated at the hospital, but will be subject to recall bias. Notably, separation of febrile seizures from acute symptomatic seizures, though useful to geneticists and epidemiologists, may remove important data. These seizures with fever could instead be studied under single underlying causes, looking for unique and shared pathogenic mechanisms.^21^

## Burden of Acute Symptomatic Seizures and Febrile Seizures

### Prevalence and incidence worldwide

The cumulative incidence (lifetime prevalence) of febrile seizures in children aged ≤6 years is 2–5% in American and European studies^2^, ^3^ and is up to 8% in Japan and 14% in Mariana Islands and Guam.^14^, ^22^ Prevalence was higher in boys than in girls in one Japanese study,^23^ whereas in an American study, it was significantly higher in African American children compared to white children.^24^ There are no population‐based data on the burden of febrile seizures in sub‐Saharan resource‐limited countries, and febrile seizures in one study^4^ are indeed acute symptomatic seizures because malaria was reported as the most important underlying cause.

The burden of acute symptomatic seizures appears to be lower than that for febrile seizures reported above. The incidence of acute symptomatic seizures per 100,000 young children was 300 in Rochester,^25^ New York, in the United States (U.S.A.) and 460 in Taiwan.^26^ The estimates were higher in males than in females both in American and Taiwanese studies. The burden of acute symptomatic seizures is highest in neonates.^25^

### Prevalence and incidence of acute symptomatic seizures in Africa

The incidence of acute symptomatic seizures in young children admitted to hospitals in Africa is in the excess of 1,000 per 100,000/year,^5^, ^6^ which is greater than estimates from Rochester (300 per 100,000/year), where case ascertainment may have been better. The incidence of acute symptomatic seizures was greatest among neonates admitted to a Kenyan hospital.^5^, ^6^, ^27^ The incidence of acute symptomatic seizures derived from hospital samples in Africa probably underestimated the true burden^5^ because only a proportion of children with acute symptomatic and febrile seizures in the community are treated at hospitals,^6^ and those with severe seizures for example, convulsive status epilepticus, may die in the community before receiving treatment of their seizures in a hospital.^28^ In a Benin study of hospitalized children,^15^ the prevalence of febrile seizures was 5% in children aged 5–60 months (the authors had referred to these as febrile seizures, but we think that some were acute symptomatic seizures caused by falciparum malaria).

One epidemiological study in Tanzania estimated prevalence of febrile seizures at 2.1%, but the use of the term “febrile seizures” did not capture acute symptomatic seizures (due to malaria or meningitis).^8^ Additionally, this study was not conducted in a demographic surveillance area, and thus vital statistics may not have been routinely updated. Finally, there were no data on risk factors for acute symptomatic seizures, and it is not clear how causes of seizures for those not hospitalized were determined. Epidemiological studies of acute symptomatic seizures and febrile seizures that differentiate between the two seizure disorders and underlying risk factors are required in Africa.

### Variation in epidemiology of acute symptomatic seizures and febrile seizures

The incidence of acute symptomatic seizures and febrile seizures varies around the world,^29^ which may be explained by several factors, including genetic susceptibility. Case definitions may differ depending on whether one follows the ILAE or the NIH definition, which have different age criteria. Case ascertainment is related to the methodology used to identify cases, where hospital registers are used in high‐income countries, but these may underestimate the number of cases in sub‐Saharan Africa, where rates of hospital use are low. Geographical variation may explain underlying risk factors; for example, poor areas in Africa that are endemic for malaria and invasive bacterial infections are likely to register more acute symptomatic seizures. Cultural factors may contribute to variation in the reported number of acute seizures by underreporting cases in areas where seizures are stigmatized, for example, in sub‐Saharan African countries.^30^

## Pathophysiology of Acute Symptomatic Seizures and Febrile Seizures: Age at Onset and Role of Underlying Causes

### Pathophysiology worldwide

The median onset of febrile seizures worldwide is usually from 18 to 24 months, but a few (up to 15%) may occur after 48 months.^31^ Fever related to a febrile illness is thought to cause the seizures, but the seizures may occur before or after the febrile illness,^32^ complicating the attribution of seizures to fever. The fact that use of antipyretic medication does not appear to prevent febrile seizures^33^ suggests that other mechanisms may be involved; for example, pro‐inflammatory molecules such as interleukins and tumor necrosis factor, which have been shown in some studies to be elevated in febrile seizures.^34^ Recently, it was shown that fever can induce hyperventilation and alkalosis,^35^ which precipitate seizures. Lower levels of iron and zinc have been reported in children with febrile seizures compared to those without, for example, in Iranian children,^36^ but their role is not fully understood. Electroencephalography (EEG) is not routinely used to study the pathophysiology of febrile seizures in high‐income countries, but neuroimaging may be performed when there is suspected neurological deficits, recurrent complex febrile seizures, or doubt about the febrile origin of the seizures.^9^

The age at onset of acute symptomatic seizures is dependent on the underlying cause, with perinatal complications, metabolic factors, and viral encephalitis important in neonates, and head injury in young children. According to ILAE,^21^ seizures with fever should be considered acute symptomatic seizures for the period until stabilization of the underlying disease, usually 1 week for head injury, 24 h for metabolic derangements, and the entire disease phase for intracranial infections. Pathogenesis for acute symptomatic seizures is insult specific; for example, hypoglycemia damages regions of the brains vulnerable to hypoxia such as the hippocampus,^37^ and this has implications in the prognosis.^38^

### Pathophysiology of acute symptomatic seizures in Africa

In Africa, onset of acute symptomatic seizures is on or before 24 months, which depends on the causes and rainfall season.^6^ Falciparum malaria can cause acute symptomatic seizures through sequestration of parasite‐infected erythrocytes in the brain microcapillaries, causing diffuse brain damage that may manifest as acute symptomatic seizures.^10^ EEGs in Kenyan children with acute symptomatic seizures showed posterior parietal‐temporal abnormalities consistent with damage to areas supplied by middle and posterior cerebral arteries.^39^ Regions supplied by these vessels are prone to hypoxia caused by falciparum malaria–related complications, particularly sequestration,^10^ severe anemia,^40^ or compromised cerebral perfusion due to hypotension and raised intracranial pressure.^41^ Falciparum malaria infection downregulates gamma amino‐butyric acid (GABA) receptors, thereby increasing susceptibility to seizures.^42^ The inflammatory molecules documented in falciparum malaria and other febrile infections^43^ may lower the seizure threshold. In addition, hyponatremia and hypoglycemia, which are common complications of malaria,^44^ may precipitate seizures. It is likely that all these mechanisms are involved in the pathogenesis of malarial acute symptomatic seizures. The role of fever in acute symptomatic seizures from malaria‐endemic areas is unclear, because most seizures occur when the child is nonfebrile,^9^ and warrants further investigation.

## Genetic Risk of Acute Symptomatic Seizures and Febrile Seizures

### Genetic susceptibility worldwide

The genetic etiology of febrile seizures is complex, with no single mode of inheritance.^45^ Heritability for seizures is high (up to 70%), and the risk of febrile seizures is up to three times in monozygotic compared to dizygotic twins, suggesting genetic susceptibility.^46^, ^47^ Six febrile seizure susceptibility loci (FEB1–FEB6) on various chromosomes have been identified (Table 2).^48^ In particular, mutations in the sodium channel and GABA genes have been identified in children with febrile seizures alone or as a broader phenotype of generalized epilepsy with febrile seizures,^49^, ^50^ suggesting febrile seizures may be related to the genes that code for ion channels.

**Table 2:** Genes associated with acute symptomatic seizures and febrile seizures

Genes	Effect (susceptibility or protective)	Study design	Country
FEB1‐6 loci	Susceptibility/protective	Linkage, GWAS	Multiple countries^48^
IL‐1Beta	Susceptibility	Case control	Finland^101^
IL‐10: rs3024500	Susceptibility	Case control	4 African sites^51^
IL‐17: rs708567	Protective	Case control	4 Africa sites^51^
G6PD: rs1050828 in females	Protective	Case control	4 Africa sites^51^
CR1‐rs17047660	Susceptibility	Case control	4 Africa sites^51^
IL‐1RN	Susceptibility	Case control	China^102^
GABRG2	Susceptibility	Case control	China^103^
CHRNA4	Susceptibility	Case control	China^104^
CSNK1G2	Susceptibility	Case control	Japan^105^
IMPA2	Susceptibility	Case control	Japan^48^
SCN1B	Susceptibility	Linkage	Australia^50^
SCN1A	Protective	Case control	Japan^106^
SCN2A	Susceptibility	Linkage	Belgium^107^

CHRNA4, cholinergic receptor nicotinic alpha‐subunit 4; CR1, complement receptor 1; CSNK1G, casein kinase 1 gamma; FEB1‐6, febrile seizures 1–6 loci; GABRG2, gamma‐aminobutyric acid type A receptor gamma‐subunit 2; G6PD, glucose‐6‐phosphate dehydrogenase; GWAS, genome‐wide association study; IL, interleukin; IMPA, inositol monophosphatase; SCN1A, sodium channel alpha‐subunit 1; SCN2A, sodium channel alpha‐subunit 2; SCN1B, sodium channel beta‐subunit 1.

### Genetic causes of acute symptomatic seizures in sub‐Saharan Africa

In sub‐Saharan Africa, only one study investigated the genetic risk of acute symptomatic seizures across four countries in Africa.^51^ This study identified two groups of polymorphisms associated with the risk of malaria‐associated acute symptomatic seizures. The first were those polymorphisms involved in the pathogenesis of severe malaria such as CR1 (Table 2), which supports the hypothesis that damage from sequestration of malaria parasites in the microvasculature of the brain precipitates the seizures. The second group comprised inflammatory molecules, in particular, interleukins, pointing to a role of fever in the acute symptomatic seizures. Other genes associated with acute symptomatic seizures, including interleukins and acetyl choline receptors, have also been identified (Table 2).

## Risk Factors for Acute Symptomatic Seizures and Febrile Seizures

### Risk factors worldwide

Risk factors for febrile seizures include developmental delay, family history of febrile seizures, neonatal care discharge after 28 days, history of day care, and some vaccinations (Table 3).^31^, ^32^ A hospital‐based study from United Arab Emirates identified duration and extent of fever as risk factors.^52^ Family history of febrile seizures is reported in up to 40% of those affected, and the risk increases with the number of family members affected.^45^, ^53^

**Table 3:** Risk factors for onset and recurrence of febrile seizures and suboptimal neurodevelopmental outcomes, including risk for epilepsy

Risk factor	Onset of febrile seizures	Recurrence of febrile seizures	Poor outcome, e.g., risk for epilepsy
Attendance at day care	Yes	Yes	No
High temperature	Yes	No	No
Prolonged stay at neonatal care	Yes	No	No
Family history of seizures	Yes	Yes	Yes
Early onset of febrile seizures	NA	Yes	No
Low temperature during seizure	NA	Yes	No
Short duration of fever	NA	Yes	No
Neurological abnormality	Yes	No	Yes
Prolonged febrile seizure	NA	No	Yes
Focal febrile seizures	NA	No	Yes
Family history of epilepsy	NA	No	Yes
Inflammatory molecules	Yes	No	Yes

NA, not applicable.

### Risk factors for acute symptomatic seizures in sub‐Saharan Africa

There are few community‐based epidemiological studies of risk for acute symptomatic seizures in Africa, and most of these studies are hospital‐based. Age is an important risk factor for acute symptomatic seizures in Africa, as evidenced by higher incidence of hospital admissions with acute symptomatic seizures in younger than older children.^5^, ^6^ The propensity of infections to cause seizures is age‐dependent, probably reflecting the idea that acquisition of natural immunity to common childhood infections such as malaria^8^ improves with age.^54^ From these hospital studies, hypoglycemia and hypoxic‐ischemic events are important risk factors of acute symptomatic seizures in the neonatal period.^5^ In a recent population‐based study in Kenya (Kariuki SM, Kombe M, Kazungu M, Odhiambo R, Bauni E, Abubakar A, Stein A & Newton CR, under preparation), which is the only epidemiological study in Africa known to investigate underlying risk factors to date, family history of febrile seizures, reported at 38%, was the most important risk factor for acute symptomatic seizures. Risk factors for acute symptomatic seizures in other low‐income settings should be identified.

## Causes of Acute Symptomatic Seizures and Febrile Seizures

### Causes worldwide

The potential causes include infections (viral, bacterial, or parasitic) and vaccinations.^45^, ^55^ The viruses mentioned in several febrile seizure studies include human herpesviruses (HHV) 6 and 7, influenza, adenovirus, respiratory syncytial virus, and parainfluenza.^56^ These viruses can account for 50% of febrile seizures in Asian countries such as Japan, Hong Kong, and China.^56^ Gastroenteritis was an important cause of febrile seizures in Turkish children,^57^ but nonfebrile seizures were also documented.^57^ Vaccines are only associated with febrile seizures in <1% of cases,^45^ and identification of two genetic loci for measles‐mumps‐rubella (MMR) vaccine‐induced febrile seizures^58^ supports a role for these vaccines in febrile seizures.

The most common causes of acute symptomatic seizures in young children from the U.S.A. include intracranial infections, such as meningitis and encephalitis; metabolic perturbations, such as electrolyte imbalance; head injury; and intracerebral hemorrhage.^25^, ^59^ In Taiwan, most acute symptomatic seizures in children were caused by gastroenteritis, intracranial infections (meningitis and encephalitis), and intracranial hemorrhage.^26^

### Causes of acute symptomatic seizures in Africa

Causes of acute symptomatic seizures in Africa are not fully understood, and there are often misconceptions about the causes, with up to 80% of Nigerian parents attributing febrile seizures to witchcraft.^60^ The most important causes of acute symptomatic seizures in these areas are falciparum malaria (up to 60%) followed by respiratory tract infections and bacterial sepsis.^6^, ^15^, ^61^ Vaccines are infrequently reported as precipitants of seizures in these settings perhaps because more attention is paid to more serious infectious causes. Viruses complicate bacterial and malarial illnesses in Africa and may have a role in acute symptomatic seizures.^17^ The role of comorbid infections in acute symptomatic seizures is, however, small because the malaria‐attributable fractions for admissions with acute symptomatic seizures was over 90%.^8^

Malaria is a common cause of fever (and therefore a potential causes of febrile seizures) but also (in a minority of cases)^8^ may involve direct central nervous system (CNS) pathology due to sequestration of parasites in the cerebral microvasculature (cerebral malaria) in what would be defined as acute symptomatic seizures.^51^ Meningitis and metabolic perturbations (hyponatremia) explain about 5% of acute symptomatic seizures admitted to hospitals, respectively,^5^, ^6^ but the proportion could be greater if there were adequate resources to perform comprehensive diagnostic investigations in Africa. There are no African estimates on acute symptomatic seizures caused by head injuries, which are studied in the context of epilepsy.^62^ Although acute symptomatic seizures occur in 5–11% of children with human immunodeficiency virus (HIV),^63^ seizures are often overlooked in the HIV studies assessing neurological outcomes.^64^ Hypoxic‐ischemic encephalopathy and sickle cell disease are associated with about 1% of acute symptomatic seizure cases admitted to hospitals in Kenya.^5^ Community‐based studies should be set up in Africa to clarify the risk factors and causes of acute symptomatic seizures in young children. These studies should also attempt to clarify the discrete categories of febrile seizures and acute symptomatic seizures in Africa to aid in fully understanding causation, management, and outcome.

## Phenotypes of Acute Symptomatic Seizures and Febrile Seizures

### Phenotypes of febrile seizures worldwide

Most febrile seizures are generalized tonic‐clonic seizures, which are short (<15 min), and few recur during the illness.^32^, ^45^ Febrile seizures in the same illness recur in up to 30% of children, are focal in up to 15%, and are prolonged in 9% of children; these are often referred to as complex seizure phenotypes.^31^, ^32^, ^45^ Febrile status epilepticus occurs in 5% of children with febrile seizures but represent about 25% of all status epilepticus in childhood.^65^ Complex phenotypes of febrile seizures (focal, repetitive, or prolonged) are associated with poorer outcomes, in particular, further prolonged attacks, epilepsy, and neurodisability.^31^, ^32^

### Phenotypes of acute symptomatic seizures in Africa

The proportion of complex (focal, prolonged, and/or repetitive) acute symptomatic seizures in Africa is twice that in high‐income countries (HICs; >60% in Kenya and elsewhere in Africa^9^, ^51^ vs. 35% in the U.S.A.^66^), but the proportions of complex acute symptomatic seizures are based on hospital data (comprising severely ill children) and could therefore be overestimated. Because complex seizures are reported in 84% of Kenyan children with nonneurological and uncomplicated malaria,^9^ it is possible these phenotypes would also be common in the acute symptomatic seizures in the community not treated at the hospital. Phenotypes such as prolonged seizures are, however, difficult to estimate in Africa because most parents in Africa do not have devices to measure time accurately.^7^, ^67^ The high incidence of complex phenotypes, however, may be ascribed to intracranial infections such as malaria, because malaria‐attributable fractions for these phenotypes were high (up to 93%).^8^ These complex seizures are associated with both localized and diffuse abnormal EEG patterns,^39^ which may influence outcome. These complex seizures can form useful phenotypes for susceptibility or protective genes^29^ and formed the bases for a multisite genetic study of acute symptomatic seizures in Africa.^51^

## Risk of Epilepsy After Acute Symptomatic Seizures and Febrile Seizures

### Risk of epilepsy worldwide

Epilepsy occurs after febrile seizures in 2–8% of children, according to reviews and population‐based studies from America and Europe,^68^, ^69^, ^70^, ^71^ although this may be a result of underlying causes rather than of seizures.^72^ The risk is higher in hospitalized febrile seizures (up to 40% develop epilepsy),^24^, ^45^ which may be biased toward severe morbidity, and in complex febrile seizures (20–40%).^71^ Generalized epilepsy is more common than focal epilepsy after febrile seizures.^73^ It is thought the risk of epilepsy is related to the mesial temporal sclerosis (MTS) following febrile seizures often documented by retrospective studies.^45^ It is unclear whether MTS is caused by febrile seizures, a preexisting MTS, or an interaction between genes and environmental factors,^74^ although a recent FEBSTAT study prospectively showed that it is related to prolonged febrile seizures.^75^

In a 10‐year follow‐up of a cohort of acute symptomatic seizures comprising 32% of children, unprovoked seizures (including those that met the criteria for the definition of epilepsy) occurred in 18%, which was 41% of those with status epilepticus and 13% of those without status epilepticus.^76^ The risk was greatest in those with status epilepticus after accounting for age, sex, and underlying causes (3.3‐fold).^76^

### Risk of epilepsy after acute symptomatic seizures in Africa

In Africa, the risk of epilepsy has been assessed only in children hospitalized with acute symptomatic seizures, but not in acute symptomatic seizures determined in the community. A recent study by Bistervels et al.,^77^ which followed 16,438 children (2,991 with acute symptomatic seizures and 13,447 without seizures), reported a twofold risk of convulsive epilepsy following acute symptomatic seizures (hazard ratio = 1.53 [95% confidence interval (CI), 1.10–2.14]), after accounting for potential confounders. Prevalence was greater in complex acute symptomatic seizures (5.9%; prevalence ratio [PR] = 1.58 [95% CI, 1.13–2.20]) or status epilepticus (7.5%; PR = 1.96 [95% CI, 1.32–2.91]) than in simple acute symptomatic seizures (3.7%). The risk was greatest for acute symptomatic seizures than for febrile seizures, suggesting that it is related to damage from underlying intracranial infections such as malaria. This finding is consistent with the elevated risk of epilepsy following cerebral malaria (in which seizures occur in up to 80%) in children from Kenya and Malawi.^78^ These studies did not perform EEG in the children with acute symptomatic seizures, so it is difficult to relate the poor outcomes with the underlying pattern of brain damage. Studies of risk of epilepsy following acute symptomatic seizures determined from the community are justified because hospital‐based acute symptomatic seizures represent severe morbidity. It is not fully understood whether EEG has any prognostic value in children with acute symptomatic seizures in the community.

## Neurocognitive Outcomes of Acute Symptomatic Seizures and Febrile Seizures

### Neurocognitive outcomes worldwide

Febrile seizures can affect neurocognitive function, particularly when seizures are complex (focal, repetitive, or prolonged). In fact, poor neurocognitive outcomes are often reported more than behavioral/emotional problems after febrile seizures.^79^ In a recent long‐term follow‐up study in China, complex febrile seizures were associated with impairment in cognitive function, but not behavioral and emotional problems.^79^ In this same study, pen and paper neurocognitive tests were more sensitive than event‐related potentials in detecting cognitive impairments. In a population‐based study in the United States, poor intelligence scores were documented in children with febrile seizures with a history of developmental delay compared to those without such history.^80^ In a recent population‐based study in Rotterdam, repetitive febrile seizures were associated with delayed language development, but not with behavioral and emotional problems and impairment in executive function.^81^ Two population‐based studies in Britain did not document poor neurocognitive and emotional/behavioral symptoms following febrile seizures.^82^, ^83^

Acute symptomatic seizures occurring during the neonatal period have poorer neurocognitive outcomes than even the febrile seizures described above. In Canada, neonatal seizures followed up for <10 years were associated with 20% risk of mental retardation and 27% risk for learning disorder.^84^

### Neurocognitive outcomes of acute symptomatic seizures in Africa

Acute symptomatic seizures occurring in the context of cerebral malaria were associated with language and cognitive impairments in Kenyan children.^85^, ^86^ Executive function was impaired in Kenyan children hospitalized with malarial seizures,^87^ which may not qualify as febrile seizures because of the potential sequestration of malaria parasites in the cerebral vasculature. Studies in Africa have assessed neurocognitive outcomes in the broader phenotype of cerebral malaria,^88^, ^89^ although acute symptomatic seizures are the most common presenting phenotype of cerebral malaria and could have a role in poor outcomes. A Kenyan study found that seizures in malaria probably resulted in memory deficits observed in cerebral malaria.^90^ Because neurocognitive impairments may be a marker of underlying neurological damage in acute symptomatic seizures, they may moderate the association between acute symptomatic seizures and behavioral/emotional problems discussed below. The lack of improvement in neurocognitive outcome following prophylaxis of seizures by phenobarbital in Kenyan children^91^ further supports the hypothesis that underlying neurological damage rather than seizures per se may determine outcome.

## Behavioral and Emotional Outcomes of Acute Symptomatic Seizures and Febrile Seizures

### Behavioral and emotional outcomes worldwide

Most studies observe that febrile seizures have a favorable prognosis in terms of neurocognitive performance and behavioral and emotional problems, although a few have documented poor outcomes. Poor behavioral and emotional problems are likely to occur in those with complex febrile seizures, underlying neurological abnormality, and increased risk of afebrile seizures.^24^ Behavioral and emotional problems after febrile seizures are more likely to occur in children with a family history of mental health problems.^92^

Behavioral and emotional problems were reported in 22% of German children with febrile seizures compared to 6% of the controls.^93^ In the United States, 18% of 120 children with febrile seizures, who did not receive prophylactic phenobarbital, developed behavioral and emotional problems, 11% of which were externalizing problems.^94^ Although the occurrence of behavioral and emotional problems in this cohort was significantly less than in those who received phenobarbital (18% vs. 42%), a drug which is known to increase hyperactivity, the results suggest a role for febrile seizures in behavioral and emotional problems. During a roundtable discussion organized by the Danish Neurological Society in 1971 in Germany, Esther Franzten reported behavioral and emotional problems such as concentration difficulties in 70/208 (34%) children with febrile seizures.^95^ Some studies in high‐income countries have not been able to replicate these results,^82^ but more studies on this subject from LMICs are justified.

### Behavioral and emotional outcomes of acute symptomatic seizures in Africa

Behavioral and emotional problems were reported in 26% of children selected from the community as controls for a case‐control study,^96^ but there is no epidemiological study in Africa examining the contribution of acute symptomatic seizures to these neurobehavioral problems. Behavioral and emotional problems occurred in approximately 10% of Kenyan children discharged with acute symptomatic seizures, although some problems may have been missed owing to spontaneous remission because this follow‐up study was conducted 9 years after discharge from the hospital.^85^ The risk for behavioral and emotional problems increased in Kenyan children previously admitted with malarial seizures (OR = 1.8),^86^ but it is unclear whether the poor behavioral and emotional outcomes were due to the seizures or malaria per se. Behavioral and emotional problems and other neurodevelopmental problems developed in a third of children with status epilepticus, 7 years after discharge from the hospital.^97^ Given that it is now possible to adapt internationally recognized behavioral tools for use in Africa,^98^ future epidemiological studies are required in Africa to clarify these findings.

## Risk Factors for Poor Outcomes Following Acute Symptomatic Seizures and Febrile Seizures

**Figure 1:**
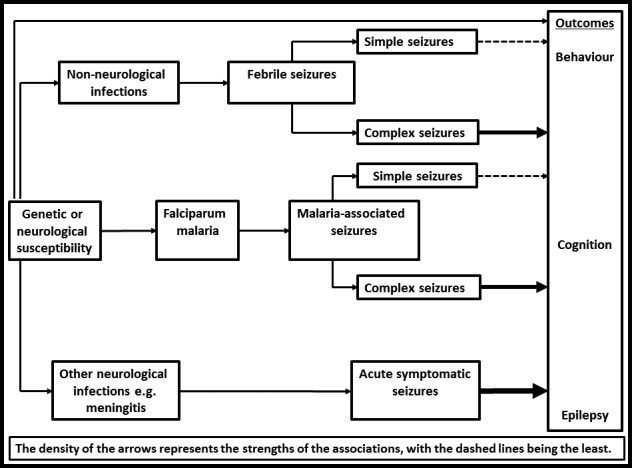
Possible factors for poor outcome of seizures with fever in children. As highlighted with the density of the arrows, acute symptomatic seizures and complex seizures are expected to be associated with poorer outcomes compared to febrile seizures and simple seizures.

Several factors increase the risk for poor mental and neurological outcomes after febrile seizures. A hospital‐based study conducted in Wales that assessed neurocognitive development but not behavior found that children hospitalized with febrile seizures had intellectual impairment if they experienced subsequent febrile seizures.^99^ In the same study, antiepileptic drugs were thought not to affect development in these children, suggesting these neurocognitive impairments may have been related to seizures.^99^ In a similar German study, behavioral problems occurred more in repetitive febrile seizures, while lower intelligence scores were associated with prolonged seizures.^93^ Kenyan children hospitalized with complex acute symptomatic seizures in malaria had worse scores for cognition and behavior persisting several years after admission.^85^ These poor outcomes are related to the type of acute symptomatic seizures, which then depends on the underlying cause/neurological damage or genetic predisposition, as outlined in Fig. 1.^51^

## Conclusions and Directions for Future Research on Acute Symptomatic Seizures in Africa

This review demonstrates that there is a paucity of population‐based studies on the prevalence of and risk factors for acute febrile seizures in sub‐Saharan Africa. Community‐based studies on acute symptomatic seizures and febrile seizures in children, particularly, young children, are therefore required to provide reliable estimates for planning purposes. Early screening of neurodevelopmental outcomes in young children is important because mortality is high in this age group, and there is a window of opportunity to initiate interventions to improve future school performance.

It is difficult to separate febrile seizures from acute symptomatic seizures in Africa because perinatal complications and intracranial infections such as falciparum malaria, viral encephalitis, and bacterial meningitis are common in the community. Any attempt to distinguish febrile seizures from acute symptomatic seizures in Africa should be informed by comprehensive investigations that rule out intracranial causes, as suggested in the ILAE guidelines. Few studies have investigated the risk factors for acute symptomatic seizures in Africa, which could be different between young children admitted to the hospital and those not hospitalized, an issue that should be investigated through community‐based studies. It is challenging to determine the causes of acute symptomatic seizures in children in the community not admitted to the hospital; recall of causes using clinical algorithms for Integrated Management of Childhood Infections may be an option, but the reliability of this method may be poor.^100^

There are little data on behavioral and emotional outcomes of young children with acute symptomatic seizures in Africa, and most available studies are hospital‐based and combine both young and older children. Few studies from developed countries that have documented poor behavioral and emotional outcomes in children with acute symptomatic seizures show a particular association with complex seizures, which are very common in Africa, where they are associated with poor outcomes, including behavioral and emotional problems. EEG may have a prognostic role in children with acute symptomatic seizures and could be routinely performed if facilities are available. Community studies examining prevalence, causes, risk factors, and behavioral and emotional outcomes of acute symptomatic seizures in young children from sub‐Saharan Africa are required to compare with other settings in the world.

## Disclosure

The authors declare no competing interests. We confirm that we have read the Journal's position on the issues involved in ethical publication and affirm that this report is consistent with those guidelines.
